# Gastrointestinal Duplication Presenting as Neonatal Intestinal Obstruction: An Experience of 15 Years at Tertiary Care Centre

**DOI:** 10.21699/jns.v5i4.432

**Published:** 2017-01-01

**Authors:** Kamal Nain Rattan, Shruti Bansal, Aastha Dhamija

**Affiliations:** 1 Department of Pediatric Surgery, Pt. B.D. Sharma PGIMS Rohtak, Haryana; 2 Department of Pathology, Pt. B.D. Sharma PGIMS Rohtak, Haryana

**Keywords:** Neonate, Gastrointestinal, Duplication cyst, Intestinal obstruction

## Abstract

Background: Gastrointestinal tract (GIT) duplications are one of the rare congenital anomalies and can occur in any portion of the gastrointestinal tract but are more commonly encountered in small intestine. The duplication cysts cause symptoms like abdominal mass and intestinal obstruction requiring surgery or may remain asymptomatic. We are reporting our 15 years’ experience duplication cysts presenting in neonates.

Methods: It is a retrospective study undertaken in the department of pediatric surgery between 2001 and 2015 for GIT duplications in neonates. Patients were analyzed for their antenatal diagnosis, age, sex, clinical diagnosis, investigatory approach, operative management and surgical outcomes.

Results: Total number of neonates, diagnosed with gastrointestinal duplication in the last 15 years, was 17. Male to female ratio was 3.3:1. The most common location was found to be the ileum occurring in 71% of cases. Apart from ileum, 2 cases of duodenal and 1 case each of gastric, colonic and cecal duplication cyst were encountered. Majority cases presented with sub-acute intestinal obstruction and were managed successfully by resection and end to end anastomosis. Associated gut atresia was found in 4 cases while 1 case was found to be associated with perforation of gut.

Conclusion: Gastrointestinal tract duplications often present with typical symptoms of gastrointestinal tract obstruction. Early diagnosis and management is required to prevent postoperative morbidity and mortality.

## INTRODUCTION

The incidence of gastrointestinal duplication is about 1 in 4500 live births and is found in 0.2% of the children. They can occur along the entire length of the gastrointestinal system. They can present at any age but 80% of cases present within first two years and majority within first three months of life with antenatal diagnosis made in significant number of cases.[1,2] Preoperative diagnosis of neonatal DC is quite challenging as to rare cause of intestinal obstruction. The objective of this study is to report our experience of 15 years in diagnosis and management of neonatal gastrointestinal duplication with focus on location, clinical presentation and associated malformations.

## MATERIALS AND METHODS

This study is a retrospective analysis of the neonates who were brought to the department of Pediatric Surgery from 2001-2015 and was diagnosed with gastrointestinal tract duplication. Data was retrieved from the patients' medical records to review for antenatal diagnosis, age, sex, clinical presentation, location, diagnostic work-up, surgical approach, associated gut malformations and post-operative course.

## RESULTS

Demography: Total number of neonates admitted with gastrointestinal duplication in the last fifteen years was 17. Amongst them, 13 were males and 4 were females accounting for male to female ratio of 3.3:1.

Presentation: All cases except one presented with abdominal lump and sub-acute intestinal obstruction. One case, however, presented with features of perforation peritonitis.

Preoperative workup: Antenatal diagnosis was made in 10 cases. A thorough investigative work up was undertaken. X ray abdomen showed multiple air fluid levels suggestive of intestinal obstruction in all patients. Ultrasonography abdomen revealed cystic lesion with dilated gut in all 17 cases. CT scan was also performed in four cases for the possibility of duplication cysts.

Management: The patients were kept nothing per oral. Nasogastric tube was placed for suction; intravenous fluids and antibiotics were given. The dehydration and electrolyte imbalance was corrected. After adequate resuscitation, the patients were operated. All the cases had solitary duplication cysts except in one case where two cysts were found. The cysts were non-communicating in all cases and their size varied greatly from smallest being 3×3cms to largest being 10×10cms in size. However, one case of giant ileal duplication cyst was encountered where the cyst measured approximately 15×12cms. One case had associated perforation of gut leading to peritonitis. Associated atresia of gut was observed in 4 cases (1 case had type III duodenal atresia, 1 had jejunal atresia with malrotation, and 2 neonates had ileal atresia). The most common site for duplication was found to be ileum present in 12 neonates, thus, accounting for 71% of cases. Two of the neonates had duodenal duplication cyst while three other neonates had gastric, colonic and cecal duplication cyst, respectively. Ileal, cecal, and colonic duplication cysts were excised along with intimately adherent part of normal GIT and end to end anastomosis was performed (Fig. 1-3). However, gastric and duodenal duplications were managed by cyst excision and mucosal stripping. The resected specimen on histopathological examination confirmed the diagnosis of gastrointestinal duplication (Fig. 4).

Follow-up: All patients had uneventful postoperative period and doing well on follow up except one patient with perforated gut which could not be saved postoperatively.

**Figure:**
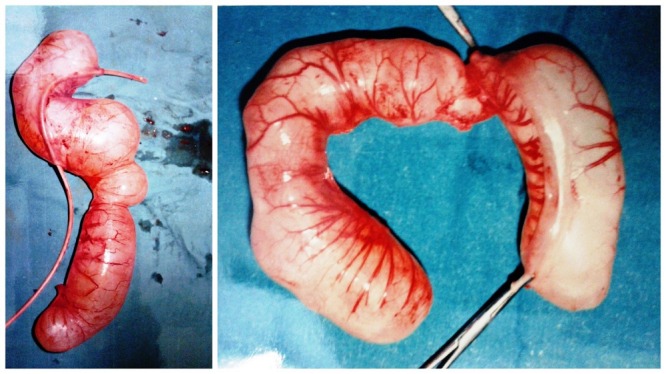
Figure 1: Gross pictures of resected ileal duplication cysts.

**Figure:**
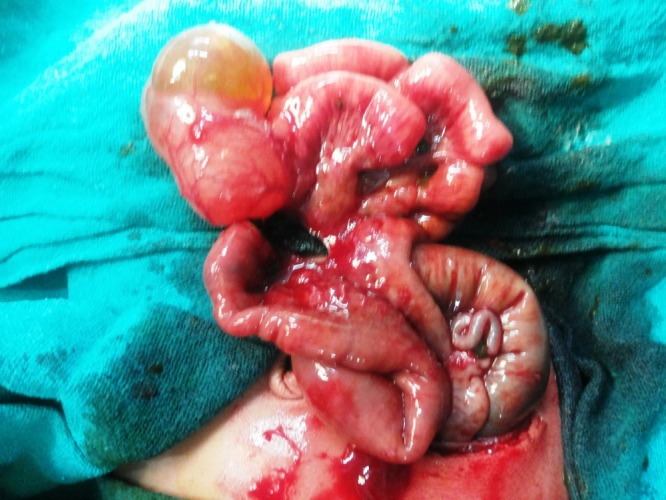
Figure 2: Peroperative photograph of ileal duplication cyst with atretic ileal segment.

**Figure:**
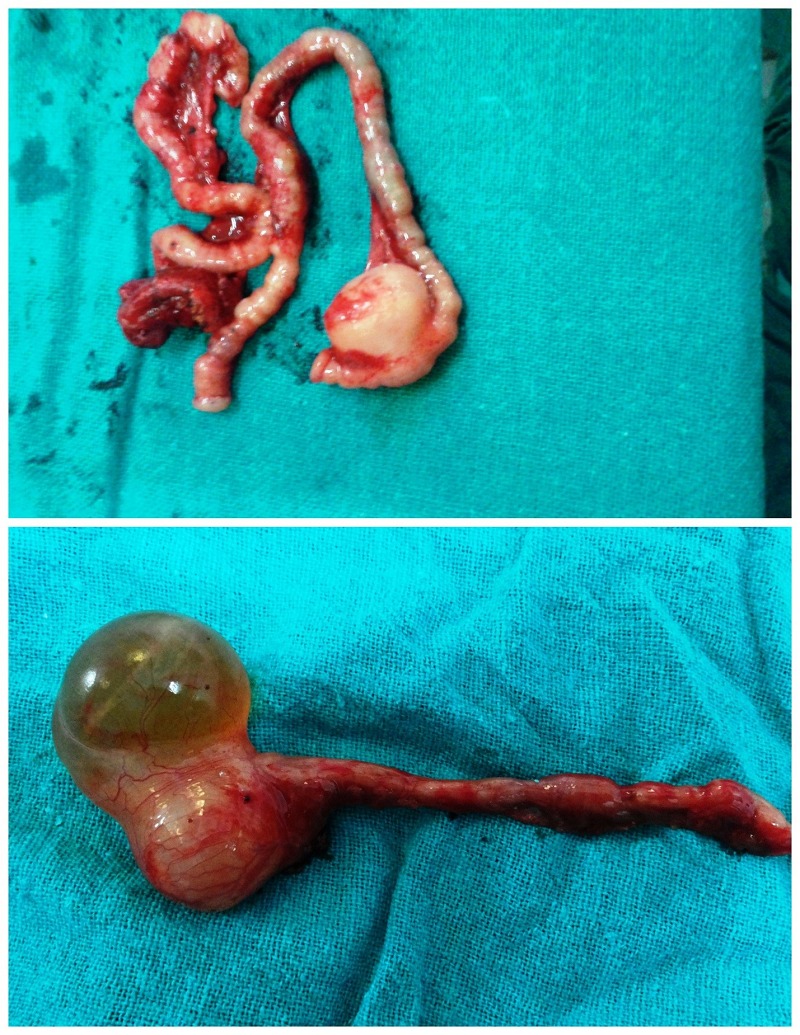
Figure 3: Resected ileal duplication cysts with atretic ileal segments.

**Figure:**
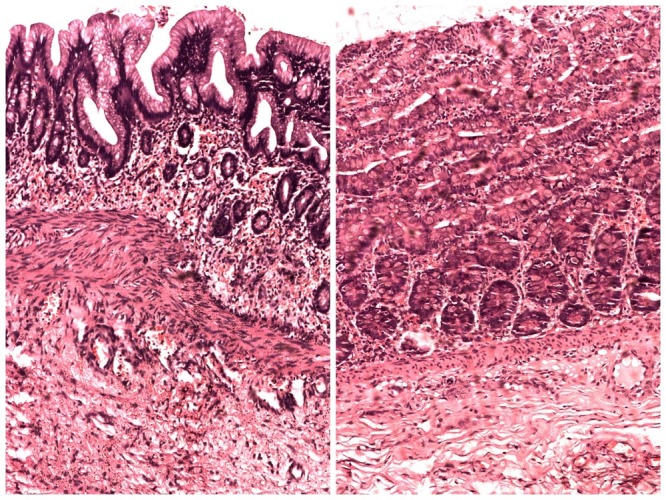
Figure 4: Photomicrograph showing histopathology of duplication cysts revealing lining of intestinal mucosa.

## DISCUSSION

Gastrointestinal duplications are the result of one or more congenital anomalies of uncertain etiology. They are generally saccular or tubular masses with presence of an intimate attachment to the GI tract, a layer of smooth muscle in the wall and an epithelial lining resembling some part of the GI tract. The cysts become incorporated into the bowel wall and share a common blood supply with the parent bowel. [1,2] Almost all duplications in our series were saccular except colonic duplication which was tubular.

Previous studies report duplications to be more common in males.[3-6] In our study also, 13 neonates were males accounting for majority of cases. The most common location reported in the literature is terminal ileum representing more than 70% of small GI duplications. Stern et al reported the incidence of duplication to be 30% in the ileum, 30% in the ileocecal calve, 8% in the stomach, 10% in the duodenum, 8% in the jejunum, 7% in the colon and 5% in the rectum. [7] In our series, maximum number of cases i.e. 13 (71%) were of ileal duplication cyst. But the site most commonly involved in our series was mid ileum rather than terminal ileum. Apart from ileum, 2 cases of duodenal duplication and 1 case each of gastric, colonic and cecal duplication cyst were encountered.

The clinical presentation of duplication cysts vary greatly from asymptomatic to acute abdomen. However, in recent days, with the increasing use of prenatal ultrasound scan, many cases are being identified in utero. Our series report 10 cases which were diagnosed antenatally. The mode of presentation usually depends on the anatomic level of the duplication, size, mass effect of lesion, presence of heterotopic gastric mucosa within the duplication and communication with the adjacent bowel and inflammation. Often they mimic other intra-abdominal conditions posing great diagnostic difficulty. Duplications can sometimes lead to complications which include perforation, intussusception, volvulus, and associated malignancy. [9] But malignancy arising from duplication cysts particularly in children is quite rare. Our majority patients presented with features of subacute intestinal obstruction. 1 case, however, was complicated by perforation of gut. Various congenital anomalies have been found to be associated with GI duplications in particular vertebral and genitourinary malformations. Although no such anomalies were found in our patients, but four cases were associated with atresia of gut. This atresia may be probably attributed to intrauterine volvulus which might have occurred as a result of duplication.

The imaging modalities commonly used to investigate duplication cysts are X-ray abdomen, ultrasonography (USG), barium studies, CT scan and magnetic resonance imaging (MRI) as they are helpful in defining the anatomic borders of a duplication. [10] USG of duplication cysts demonstrate an echogenic inner mucosal layer and a hypoechoic outer muscular layer - “double-wall” sign which is highly indicative of enteric duplications and thus USG is considered to be the first choice imaging modality. [11] A contrast study demonstrates a submucosal mass with mass effect extending into the lumen of the gastrointestinal tract. CT scan and MRI are not used routinely but are quite helpful in difﬁcult cases. Radionuclide scanning with technetium-99m sodium pertechnetate can be used in cases with suspicion of presence of heterotopic gastric mucosa.

The treatment of choice for GIT duplication remains surgical excision taking into account the location, size and benign nature of lesion. The aim of the intervention when possible should be total resection as partial excision is associated with high risk of recurrence. [12] We treated all our cases with surgical resection and end to end anastomosis except gastric and duodenal duplication where cyst excision was performed with mucosal stripping. In the last two decades, minimally invasive surgery has gained much importance in the field of pediatric abdominal surgery. But literature review reveals only few controlled studies of the outcome in laparoscopic surgery in alimentary tract duplications. Shehi et al attempted laparoscopic excision of gastric duplication cyst in a neonate but they had to convert it to open laparotomy owing to the difficulty to separate the cyst from the stomach. [13] However, few cases are reported in the literature which were successfully treated laparoscopically. [14]

## CONCLUSION

GIT duplications, albeit rare, are still not uncommon in the pediatric population. The clinical presentation is quite diverse and often mimics other intra-abdominal conditions thus posing a great challenge to pediatric surgeons to arrive at clinical diagnosis preoperatively. Therefore high index of suspicion should be kept in mind when dealing with neonates presenting with features of intestinal obstruction. Overall, open surgery is safe with good tolerance rates but minimally invasive surgery should be considered in centers with adequate expertise.

## Footnotes

**Source of Support:** None

**Conflict of Interest:** None
